# Empfehlungen der DGRh-Kommission für Komplementäre Heilverfahren und Ernährung zur Phytotherapie: Ergänzung zur Anwendung von ausgewählten Phytotherapeutika und pflanzlichen Präparaten in der Rheumatologie: Erweiterung um 13 Heilpflanzen

**DOI:** 10.1007/s00393-026-01840-2

**Published:** 2026-07-14

**Authors:** G. Keyßer, Oliver Sander, Inna Frohne, Alexander Pfeil, Andreas Michalsen, Olaf Schultz, Monika Reuß-Borst, Olga Seifert

**Affiliations:** 1 Klinik und Poliklinik für Innere Medizin II, Universitätsklinikum Halle (Saale), Ernst-Grube-Straße 40, 06120 Halle (Saale), Deutschland; 2 Klinik für Rheumatologie, Universitätsklinikum Düsseldorf, Moorenstr. 5, 40225 Düsseldorf, Deutschland; 3 Privatpraxis für Rheumatologie und ganzheitliche Medizin, Frankenstraße 238, 45134 Essen, Deutschland; 4 Klinik für Innere Medizin III, FB Rheumatologie/Osteologie, Universitätsklinikum Jena, Am Klinikum 1, 07740 Jena, Deutschland; 5 Immanuel Krankenhaus Berlin, Königstraße 63, 14109 Berlin, Deutschland; 6 Acura Kliniken Baden-Baden, Rotenbachtalstraße 5, 76530 Baden-Baden, Deutschland; 7 Schwerpunktpraxis Innere Medizin, Frankenstraße 36, 97708 Bad Bocklet, Deutschland; 8 Klinik und Poliklinik für Rheumatologie, Universitätsklinikum Leipzig, Liebigstr. 20, Haus 4, 04103 Leipzig, Deutschland

**Keywords:** Komplementäre Heilverfahren, Heilpflanzen, Rheumatische Erkrankungen, Degenerative Gelenkerkrankungen, Nahrungsergänzungsmittel, Complementary treatment, Medicinal plants, Rheumatic diseases, Degenerative joint diseases, Nutritional supplements

## Abstract

**Einleitung:**

Heilpflanzen und ihre Zubereitungen stoßen bei vielen Patienten mit rheumatischen Erkrankungen auf Interesse. Die DGRh-Kommission für Komplementäre Heilverfahren und Ernährung (KHE) hat im Jahre 2023 damit begonnen, die wissenschaftliche Literatur zu ausgewählten Phytotherapeutika und pflanzlichen Präparaten zu sichten und Möglichkeiten für ihre Anwendung in der Rheumatologie zu prüfen. Nachdem 2025 erste Ergebnisse veröffentlicht wurden, folgte im gleichen Jahr die Begutachtung von 13 weiteren Phytotherapeutika.

**Methoden:**

In einer Online-Sitzung der Kommission KHE am 07.01.2025 wurde eine weitere Liste von Phytotherapeutika erstellt, die in der Rheumatologie häufig Anwendung finden (meist als Selbstmedikation). Mitglieder der Kommission führten daraufhin Literaturrecherchen zu diesen Präparaten durch und fassten die Ergebnisse nach einer vorab vereinbarten Matrix zusammen. Dabei wurde zu Beinwell, Bergwohlverleih (Arnica), Curcuma, Ingwer, Katzenkralle (Uncaria tomentosa), Knoblauch, Kohlblättern, Mariendistel, Pycnogenol (See-Kiefer-Rindenextrakt), Schachtelhalm, Teufelskralle (Harpagophytum), Weihrauch (Boswellia) und Wilfords Dreiflügelfrucht (Tripterygium wilfordii) recherchiert. Die Ergebnisse wurden im Umlaufverfahren überprüft, in einer Online-Sitzung der Kommission KHE konsentiert und vom Vorstand der Deutschen Gesellschaft für Rheumatologie und klinische Immunologie (DGRh) final bestätigt.

**Ergebnisse:**

Für alle untersuchten Pflanzenstoffe liegen Berichte über entzündungshemmende oder immunologische Effekte in vitro und/oder im Tiermodell vor. Die Evidenz für einen klinisch relevanten Nutzen ist jedoch sehr spärlich. Für keines der untersuchten Präparate kann eine Anwendung bei entzündlichen Gelenkerkrankungen empfohlen werden. Wenn Arnica, Beinwell, Curcuma, Ingwer, Knoblauch, Kohlblätter, Pycnogenol, Teufelskralle oder Weihrauch bei degenerativen Gelenkerkrankungen auf Patienten-Initiative eingenommen werden, müssen Rheumatologen nicht abraten, wenn ansonsten ein sinnvolles Therapiekonzept eingehalten wird. Ingwer, Knoblauch und Curcuma in natürlicher Form können als Lebensmittel im Rahmen einer gesundheitsbewussten Ernährung empfohlen werden.

**Schlussfolgerung:**

Auch die wissenschaftliche Analyse einer zweiten Serie von pflanzlichen Präparaten hat gezeigt, dass der belegbare Stellenwert der Phytotherapie für das Fachgebiet gering ist. Dabei müssen die hier vorgestellten pflanzlichen Präparate für die rheumatologische Praxis jedoch differenziert betrachtet werden.

## Abstract (en)

**Introduction:**

Medicinal plants and their preparations are of great interest to many patients with rheumatic diseases. In 2023 the German Society for Rheumatology and Clinical Immunology (DGRh) Committee for Complementary Medicine and Nutrition (KHE) began reviewing the scientific literature on selected phytotherapeutic agents and herbal preparations and examining their potential applications in rheumatology. Following the publication of the initial results in 2025, the evaluation of 13 additional phytotherapeutic agents took place later that same year.

**Methods:**

In an online meeting of the KHE Committee on 7 January 2025, a further list of phytotherapeutic agents frequently used in rheumatology (mostly as self-medication) was compiled. Committee members then conducted literature searches on these herbal preparations and summarized the results according to a predefined matrix. Research was conducted on comfrey, arnica, turmeric, ginger, cat’s claw (Uncaria tomentosa), garlic, cabbage leaves, milk thistle, Pycnogenol (extract from the bark of maritime pine), horsetail, devil’s claw (Harpagophytum), frankincense (Boswellia) and Wilford’s tripter fruit (Tripterygium wilfordii). The results were reviewed using a circulation procedure, a consensus was agreed upon in an online meeting of the KHE committee and finally confirmed by the German Society for Rheumatology and Clinical Immunology (DGRh).

**Results:**

For all plant substances investigated, there are reports of anti-inflammatory or immunological effects in vitro and/or in animal models; however, the evidence for a clinically relevant benefit is very limited. None of the investigated preparations can be recommended for use in inflammatory joint diseases. If arnica, comfrey, turmeric, ginger, garlic, cabbage leaves, Pycnogenol, devil’s claw or frankincense are taken at the patient’s own initiative for degenerative joint diseases, rheumatologists do not need to advise against them, provided a sensible treatment plan is otherwise being followed. Ginger, garlic and turmeric in their natural form can be recommended as foods within the framework of a health-conscious diet.

**Conclusion:**

The scientific analysis of a second series of herbal preparations has also shown that the confirmed value of phytotherapy for the field is limited; however, for the rheumatological practice a differentiated consideration is needed for the herbal preparations presented here.

Ungeachtet aller Fortschritte in der Rheumatherapie ist das Interesse von Patientinnen und Patienten mit entzündlich-rheumatischen Erkrankungen an pflanzlichen Präparaten nach wie vor hoch. Auch die Zahl wissenschaftlicher Beiträge zum Thema „herbal medicine“ und „arthritis“ steigt von Jahr zu Jahr an. So wurden seit Anfang 2025 mehr als 600 neue Publikationen zu diesen beiden Stichworten in die PubMed-Datenbank der National Library of Medicine eingestellt.

Die Kommission für Komplementäre Heilverfahren und Ernährung (KHE) hat 2025 eine erste Bewertung von traditionellen Phytopharmaka und Nahrungsergänzungsmitteln (NEM) publiziert [1]. Dabei wurden Literaturrecherchen zu Borretsch-Öl, Brennnesselpräparaten, Cannabis-Präparaten sowie Zubereitungen von Rosa canina, Rosmarin, Safran und Weidenrinde sowie zum Mischpräparat Phytodolor® durchgeführt. Die Evidenz für einen klinisch relevanten Nutzen in der Rheumatologie wurde als spärlich beurteilt und eine Anwendung bei entzündlichen Gelenkerkrankungen nicht empfohlen. Allerdings hält es die Kommission KHE für wenig realistisch, an die Bewertung der Studienlage zu pflanzlichen Präparaten die gleichen strengen Maßstäbe anzulegen wie an Pharmaka, die eine reguläre Arzneimittelzulassung durchlaufen haben. Da phytotherapeutische Präparate oft auf Eigeninitiative der Patienten eingenommen werden, führte die Kommission KHE in Bezug auf degenerative Gelenkerkrankungen die Bewertungskategorie „keine aktive Empfehlung“ ein. Diese Kategorie betraf Präparate auf Basis von Brennnessel, Weidenrinde oder Rosa canina, sowie das Mischpräparat Phytodolor®: Wenn diese bei degenerativen Gelenkerkrankungen auf Patienten-Initiative eingenommen werden, müssen Rheumatologen nicht abraten, wenn ansonsten ein sinnvolles Therapiekonzept eingehalten wird [1].

Unmittelbar nach Erscheinen der ersten Veröffentlichung wurde an die Kommission KHE der Wunsch nach der Bewertung von weiteren pflanzlichen Substanzen herangetragen. Daher wurde eine zweite Liste von häufig eingenommenen pflanzlichen Präparaten erarbeitet und bewertet.

## Methodik

Mitglieder der Kommission KHE erstellten in einer Online-Sitzung am 07.01.2025 eine weitere Liste zu ausgewählten frei verkäuflichen pflanzlichen Präparaten und Phytotherapeutika. Jedes Mitglied der Kommission führte eine Literaturrecherche zu einem oder zwei Präparaten durch, analog zum Vorgehen der ersten Liste [1].

Recherchiert wurde zu Beinwell, Bergwohlverleih (Arnica), Curcuma, Ingwer, Katzenkralle (Uncaria tomentosa), Knoblauch, Kohlblättern, Mariendistel, Pycnogenol (einem Extrakt aus der Rinde der See-Kiefer Pinus pinaster), Schachtelhalm, Teufelskralle (Harpagophytum), Weihrauch (Boswellia) und Wilfords Dreiflügelfrucht (Tripterygium wilfordii). Die Ergebnisse wurden im Umlaufverfahren überprüft und in einer weiteren Online-Sitzung konsentiert. Anschließend wurden die Ausarbeitungen dem Vorstand der Deutschen Gesellschaft für Rheumatologie (DGRh) übermittelt und von diesem kritisch gewürdigt. Nach letztmaliger Überarbeitung wurden die Empfehlungen ab März 2026 online zugänglich gemacht.

## Ergebnisse

Die ausführlichen Versionen der Literaturrecherchen sowie der daraus abgeleiteten Empfehlungen und die vollständigen Literaturverzeichnisse sind auf der Homepage der DGRh abrufbar: https://www.dgrh.de/lesen/empfehlungen/komplementaere-ansaetze/. Diese Ausarbeitungen der Kommission werden hier in stark verkürzter Form wiedergegeben. Eine Übersicht über die Empfehlungen liefert Tab. 1.

**Tab. 1:** Empfehlungen der KHE zur Phytotherapie. Keine *aktive* Empfehlung: Bei Wunsch der Patienten nach Selbstmedikation muss nicht abgeraten werden.

Präparat	Vermutete Wirksubstanzen	Anwendung bei entzündlichen Gelenkerkrankungen	Anwendung bei Arthrosen	Sicherheitsaspekte/Sonstiges
Beinwell	Allantoin, Phenolsäuren Naphtoquinone und Flavonoide	Keine Empfehlung	Keine *aktive* Empfehlung	Pflanze enthält Pyrrolizidinalkaloide, die potenziell leberschädigend und kanzerogen sind
Bergwohlverleih Arnica montana	Sesquiterpenlactone Flavone und ätherische Öle, Triterpene und Phenolcarbonsäuren	Keine Empfehlung	Keine *aktive* Empfehlung	Mögliche Kontaktdermatitis bei topischer Anwendung, orale Präparate nicht bei Schwangerschaft und Stillzeit
Curcuma	Curcumin, Demethoxycurcumin, Bisdemethoxycurcumin	Keine Empfehlung	Keine *aktive *Empfehlung	Präparate mit verbesserter Bioverfügbarkeit: noch keine Belege über Wirksamkeit und Sicherheit. Empfehlung zur Aufnahme als Gewürz im Rahmen einer gesundheitsbewussten Ernährung
Ingwer	Gingerole, Shogaole, Paradole und Zingeron	Keine Empfehlung	Keine *aktive* Empfehlung	Noch unsichere Datenlage zu Interaktionen mit Substanzen, die die Thrombozytenaggregation hemmen. Empfehlung zur Aufnahme im Rahmen einer gesundheitsbewussten Ernährung
Knoblauch	Allicin (Allyl 2‑propenethionsulfinat oder Diallylthiosulfinat), Allylsulfide, Diallylsulfide, Vinyldithiine und Ajoene	Keine Empfehlung	Keine Empfehlung	Vereinzelt Asthma und allergische Reaktionen. Empfehlung zur Aufnahme im Rahmen einer gesundheitsbewussten Ernährung
Kohlblätter	Glucosinolate (Senföle), Flavonoide, Anthocyane, phenolische Verbindungen	Keine Empfehlung	Keine *aktive* Empfehlung	–
Mariendistel	Silibinin, Isosilibinin, Silichristin und Silidianin, Flavonoide und polyphenolische Substanzen	Keine Empfehlung	Keine Empfehlung	Vereinzelt Erhöhung von Bilirubin und Leberenzymen
Pycnogenol	Polyphenole, oligomere Proanthocyanidine (OPC), Bioflavonoide, organische Fruchtsäuren	Keine Empfehlung	Keine *aktive *Empfehlung	–
Schachtelhalm	Kieselsäure, Flavonoide, Kaffeesäurederivate	Keine Empfehlung	Keine Empfehlung	–
Teufelskralle	Harpagosid, Flavonoide, Phenylethanolglykoside, Phytosterine, Triterpene und Zimtsäure	Keine Empfehlung	Keine *aktive *Empfehlung	Vereinzelt gastrointestinale Unverträglichkeit, allergische Reaktionen (Urtikaria), keine Erfahrungen in Schwangerschaft und Stillzeit
Tripterygium wilfordii	Triptolid und Celastrol	Keine Empfehlung	Keine Empfehlung	Nicht selten Anstieg der Leberenzyme, gastrointestinale Unverträglichkeit
Uncaria tomentosa	Oxindol-Alkaloide, Terpenoide, Sterole, Flavonoide und Hydroxycarbonsäuren	Keine Empfehlung	Keine Empfehlung	Einzelne Fälle von interstitieller Nephritis, Arzneimittelinteraktionen nicht ausgeschlossen
Weihrauch	11-keto-β-Boswellinsäure (KBA) und acetyl-11-keto-β-Boswellinsäure (AKBA)	Keine Empfehlung	Keine *aktive* Empfehlung	–

## Beinwell

Beinwell gehört zu den Raublattgewächsen (Boraginaceae). Als Heilpflanze wird Symphytum officinale angebaut. Potenziell aktive Bestandteile sind Allantoin (ein Imidazolidin-Derivat), Phenolsäuren (Rosmarinsäure, Hydroxyzimtsäure), Naphthoquinone (Shikonin) und Flavonoide. Beinwell findet sich verbreitet in der Kulturlandschaft. Genutzt wird überwiegend die Wurzel.

Beinwellpräparate werden als Externa, Tropfen und Globuli vertrieben. Im MMI Pharmaindex sind 8 Wirkstoffe gelistet (z. B. Beinwell-Blätter-, -Blüten-, -Wurzel-Extrakt, Beinwellkraut-Presssaft [3–8:1]). Als apothekenpflichtiges Arzneimittel steht Beinwell als Kapsel oder als Schmerzsalbe zur äußerlichen Behandlung von Muskel- und Gelenkschmerzen infolge stumpfer Verletzung, von Kniegelenkarthrose oder Rückenschmerzen zur Verfügung.

Eine PubMed-Suche nach Beiträgen zum Stichwort „Comfrey“ lieferte 2 Analysen klinischer Studien bei Verletzungen, Gonarthrose und Rückenschmerz, wobei die Qualität der Studien nicht befriedigend war [2–4], jedoch keine Studien zur Wirksamkeit bei Patienten mit entzündlich-rheumatischen Erkrankungen. Bei einer Studie mit 220 PatientInnen mit Gonarthrose wurde eine Schmerzlinderung ohne einen Anstieg an Nebenwirkungen im Vergleich mit Placebo beobachtet. Über einen Einfluss auf die Gelenkfunktion wurde nicht berichtet [5]. Eine Empfehlung zu topischen Phytotherapeutika konnte in der aktuellen deutschen Leitlinie Gonarthrose nicht gegeben werden.

Beinwell enthält Pyrrolizidinalkaloide, die leberschädigend und krebsauslösend sein können. Die Tageshöchstdosis von Pyrozolinen wird mit 100 mg benannt, Dosen von 10–100 mg sollten auf 4–6 Wochen/Jahr beschränkt bleiben.^1^ Neue, gereinigte Präparate liegen bei sachgemäßer Anwendung unter der als unbedenklich eingestuften Dosis von 10 mg/Tag und sind damit nicht eingeschränkt. Dennoch sollte eine besondere Nutzen- und Risikoabwägung bei Kindern und Schwangeren erfolgen.

Die wissenschaftliche Evidenz reicht nicht aus, um Beinwellpräparate für Patienten mit einer entzündlich-rheumatischen Krankheit zu empfehlen. Bei Behandlungswunsch auf Seiten der Patienten aufgrund von Arthroseschmerzen, Verstauchungen und unspezifischen Rückenschmerzen muss von einer selbstinitiierten Behandlung mit den im Handel befindlichen Cremes aus Beinwellwurzel-Präparaten nicht abgeraten werden.

## Bergwohlverleih (Arnica montana)

Die Echte Arnika (Arnica montana) bekannt als Bergwohlverleih, ist eine europäische Gebirgspflanze aus der Familie der Korbblütler (Asteraceae). Sie gilt als Giftpflanze. Hauptwirkstoffe der Arnikablüten sind Sesquiterpenlactone, insbesondere Helenalin- und Dihydrohelenalin-Ester. Daneben enthalten die gelben Blütenkörbe Flavone, Flavonole und ätherisches Öl sowie Phenolcarbonsäuren (https://de.wikipedia.org/wiki/Arnika).

Helenalin inhibiert in vitro die NF-κB- und Telomeraseaktivität, beeinträchtigt die Protein- und DNA-Synthese und induziert in aktivierten CD4+-T-Zellen-Apoptose. Arnikatinktur hemmt in Zellkulturmodellen TNF‑α, IL‑6, COX‑2, MCP‑1 und ICAM‑1 [6]. In der Kollagen-induzierten Arthritis von Ratten reduzierte die orale Gabe von Arnika-Blütenmethanolextrakt klinische Symptome und verbesserte die Gelenkfunktion [7].

In Deutschland sind etwa 200 Zubereitungen mit Arnika verfügbar, darunter zahlreiche homöopathische Präparate. Als rezeptfreie Arzneimittel werden mehrere Arnikapräparate angeboten, z. B. als Tinktur, Kapseln, Salbe und Wundtücher.

Eine PubMed-Suche zum Stichwort „Arnica montana“ lieferte 420 Literaturstellen, zum Stichwort „Helenalin“ 185. Davon sind 62 klinische Studien. Diese betreffen v. a. postoperative Schmerzen, Ödem, Wundheilung nach orthopädischen, zahnärztlichen, plastisch-chirurgischen Angriffen sowie degenerative Gelenkveränderungen. Eine Metaanalyse von 28 Studien untersuchte den Einfluss von Arnika vor oder nach Operationen auf Wundheilung, Blutungen, Schwellungen oder Schmerzen. Vergleiche mit Placebo ergaben unbedeutende und nicht signifikante Effekte. Studien mit aktiven Vergleichspräparaten (NSAR, Paracetamol) zeigten eine heterogene, statistisch signifikante, aber geringe Effektstärke [8]. Eine offene, unkontrollierte Studie untersuchte die Anwendung eines Arnicagels bei Patienten mit leichter bis mittelschwerer Kniearthrose. Nach 6 Wochen verbesserte sich der Western Ontario and McMaster Universities Osteoarthritis Index (WOMAC-Score) hinsichtlich Schmerz, Steifigkeit und Funktion signifikant. Die Behandlung zeigte lediglich geringe lokale Nebenwirkungen [9]. Eine randomisierte Doppelblindstudie bei Fingerpolyarthrose hat nach 21 Tagen keinen Unterschied bezüglich Schmerz und Funktionseinschränkung zwischen Ibuprofen (5 % Gel) oder Arnika (50 g Tinktur/100 g Gel) gezeigt [10]. Weitere Studien weisen methodische Schwächen auf, wie kleine Stichprobengrößen oder fehlende Placebokontrollen. Daten zur Behandlung mit Arnika bei entzündlich-rheumatischen Erkrankungen sind nicht vorhanden.

Die orale Einnahme von Arnikaprodukten sollte aufgrund toxischer Inhaltsstoffe vermieden werden [11]. Die Dosierung ist nicht für alle Produkte standardisiert. Der Wirkstoffgehalt der Pflanzen kann standortbedingt schwanken, woraus unterschiedliche Konzentrationen der Inhaltsstoffe zwischen verschiedenen Herstellern resultieren können [12]. Arnikasalbe kann eine Kontaktdermatitis bewirken [13]. Unter oraler Einnahme wurden gastrointestinale Nebenwirkungen, Kopfschmerzen und Schläfrigkeit beobachtet [14]. Die Anwendung von Arnika gemeinsam mit Antikoagulanzien oder Thrombozytenaggregationshemmern sowie während Schwangerschaft und Stillzeit gilt als unsicher [15]. Es fehlen Daten zur Sicherheit bei langfristiger Anwendung, insbesondere bei höheren Dosierungen.

Die Anwendung von Arnica montana aufgrund eines entzündlich-rheumatischen Krankheitsbildes kann nicht empfohlen werden. Von der topischen Anwendung bei Patienten mit Arthrose muss bei Therapiewunsch des Patienten nicht abgeraten werden.

## Curcuma

Die Indische Gelbwurz oder Kurkuma (lat. Curcuma longa) stammt aus der Familie der südostasiatischen Ingwergewächse. Als Kurkuma wird das orangegelbe Pulver aus den Wurzelstöcken bezeichnet. Hauptbestandteil ist Curcumin [16]. Im MMI Pharmindex sind ca. 200 Präparate mit Curcuma aufgeführt, entweder als Monosubstanz oder in Kombination mit anderen Pflanzenstoffen wie schwarzem Pfeffer. Die Mehrzahl wird als nichtapothekenpflichtige NEM angeboten, mindestens 50 als homöopathische Präparate.

Curcumin ist ein natürlich vorkommendes Diarylheptanoid aus der Stoffgruppe der pflanzlichen Polyphenole. Die Substanz ist schlecht und inkonsistent bioverfügbar. Hilfsstoffe wie Piperin, ein Alkaloid aus schwarzem Pfeffer, oder Detergentien sollen die Aufnahme von Curcumin verbessern. In vitro weist Curcumin immunmodulierende und antiinflammatorische Effekte auf. Die antioxidative Wirkung beruht u. a. auf der Hemmung von Cyclooxygenasen und Lipoxygenasen. In mehreren Arthritis-Tiermodellen wirkte Curcumin antientzündlich und antierosiv.

Curcumin ist als NEM in zahlreichen Präparaten in Deutschland erhältlich. Es existiert bisher kein pharmakologisch eindeutig definiertes, geprüftes und zugelassenes Arzneimittel auf der Basis von Curcuma auf dem deutschen Markt.

Eine Literaturrecherche zu den Stichworten „turmeric“ und „arthritis“ lieferte für Untersuchungen am Menschen 159 Zitate. Davon erschienen 28 als „clinical trial“. Unter „curcumin“ und „arthritis“ wurden 296 Zitate aufgeführt. Die Mehrzahl der Studien zu Curcuma wurden an indischen oder thailändischen Arthrosepatienten durchgeführt, überwiegend mit Gonarthrose [17–26]. Verwendet wurde Curcumin als Reinextrakt oder in Formulierungen, die auf eine Verbesserung der Bioverfügbarkeit gerichtet waren [21–23, 27, 28]. Zur Anwendung kamen auch Kombinationen, u. a. mit Weihrauch (Boswellia serrata), Schlafbeere (Withania somnifera) oder Zink. Berichtet wurde über eine stärkere Wirkung als Placebo [21, 27, 29], Gleichwertigkeit zu NSAR [26], Paracetamol [23] oder Standardbehandlung [28]. Eine Metaanalyse von 15 randomisierten kontrollierten Arthrosestudien mit 1621 Patienten bescheinigte Curcuma-longa-Extrakt und Curcumin eine positive Wirkung auf die visuelle Analogskala für Schmerz und die WOMAC-Scores für Schmerz, Funktion und Steifheit bei guter Verträglichkeit. Die nicht mehr aktuelle deutsche S3-Leitlinie zur Coxarthrose von 2019 erwähnt positive Studien zu Curcuma bei degenerativen Gelenkerkrankungen, hält die Studienlage für eine Empfehlung aber für unzureichend^2^, in der überarbeiteten Version von 2021 wird Curcuma nicht mehr erwähnt.

Für die RA existiert eine dreiarmige, doppelblinde Studie an je 12 Patienten mit Curcumin in einer Formulierung mit erhöhter Bioverfügbarkeit. Die Ergebnisse zeigten eine hochsignifikante Abnahme des DAS28 nach 90 Tagen, die mit 53 bzw. 66 % deutlich über den Effekt einer Biologika- oder JAK-Inhibitoren-Therapie hinausgehen würde, während in der Placebogruppe keine Verbesserung auftrat [30]. Die Studie offenbart jedoch ausgeprägte Schwächen, welche die Plausibilität der Daten in Frage stellen.

Eine zweite, einfachblinde Pilotstudie an je 3 Gruppen zu 15 Patienten verglich eine Therapie mit 2 × 500 mg Curcumin mit erhöhter Bioverfügbarkeit mit 2 × 50 mg Diclofenac und einer Kombination beider Präparate über 8 Wochen [31]. Curcumin wurde als effektiver und verträglicher im Vergleich zu Diclofenac beschrieben. Auch in dieser Studie gingen die Response-Raten in der Curcumin-Monotherapiegruppe deutlich über das von Biologika bekannte Niveau hinaus. Eine systematische Literaturrecherche attestierte beiden Studien ein hohes Risiko für Bias [32]. In einer weiteren randomisierten Studie wurden 65 Patienten entweder mit Placebo oder Curcumin als Nanomicellen behandelt. Nach 12 Wochen zeigte der DAS28 eine nichtsignifikante, tendenziell stärkere Abnahme unter Verum [33].

Die Europäische Food Safety Authority (EFSA) empfiehlt als akzeptable tägliche Aufnahmemenge („acceptable daily intake“, ADI) von Curcuma als Lebensmittelzusatzstoff E100 ein Quantum von 3 mg pro kg Körpergewicht [34]. Die bisherigen Daten zu nativem Curcumin zeigen, dass in Studien Tagesdosen von bis 8 g keine relevanten Nebenwirkungen zeigten (Übersicht in [35]). Curcumin in Form von Nanopartikeln wies auch in hohen Dosen keine relevante akute, chronische oder Genotoxizität auf [36].

Das Bundesamt für Verbraucherschutz und des BfArM^3^ stellt zu Curcuminprodukten mit verbesserter Bioverfügbarkeit fest, dass Curcumazubereitungen als traditionelles pflanzliches Arzneimittel eingestuft werden könnten, sofern ein Bezug zu bestimmten Erkrankungen hergestellt wird. „Für eine Einstufung als nichttraditionelles Arzneimittel wären Nachweise über Wirksamkeit und Sicherheit zu erbringen; zurzeit liegen jedoch keine ausreichenden klinischen Belege dafür vor.“

Trotz zahlreicher Hinweise auf entzündungshemmende, antioxidative und immunmodulierende Eigenschaften von Curcumin in vitro und im Tiermodell reicht die Datenlage nicht aus, um eine Anwendung von Curcumin oder Curcuminpräparaten mit verbesserter Bioverfügbarkeit bei entzündlichen Gelenkerkrankungen oder Autoimmunerkrankungen zu empfehlen. Patienten mit degenerativen Gelenkerkrankungen, welche Curcuma auf eigenen Wunsch als NEM mit der Erwartung positiver Effekte auf ihre Symptome konsumieren, muss von einer Anwendung nicht abgeraten werden. Natürliches Curcuma als Gewürz wird als sinnvoller Bestandteil einer pflanzenbasierten, entzündungshemmenden Ernährung angesehen.

## Ingwer

Ingwer aus der Familie der Zingiberaceae wird seit über 3000 Jahren als Gewürz und Heilpflanze genutzt. Seine pharmakologischen Eigenschaften sind auf v. a. auf Phenole und Terpene zurückzuführen [37]. Die Rhizome enthalten Oleoresin für den scharfen Geschmack und ätherische Öle. Das Oleoresin beinhaltet physiologisch aktive Substanzen wie Gingerole, Shogaole, Paradole und Zingeron [38]. Die ätherischen Öle enthalten hauptsächlich Sesquiterpene wie α‑Zingiberen. Einer der Hauptbestandteile von Ingwer, das 6‑Gingerol, wirkt entzündungshemmend und schmerzlindernd und verleiht dem Ingwer die Schärfe [39].

Shogaole wirken in vitro antioxidativ sowie antiinflammatorisch und antikanzerogen [40–42].

Die Lagerung, thermische Verarbeitung, Trocknungs- und Extraktionsmethoden beeinflussen die Zusammensetzung des Ingwers. Standardisierte Präparate auf dem deutschen Markt werden zur Vorbeugung von Reisekrankheit wie Schwindel, Übelkeit und Erbrechen angewendet. Als NEM sind von verschiedenen Herstellern Ingwerkapseln mit Ingwerwurzelpulver oder Ingwerwurzelextrakt (Dosierung 500–650 mg) verfügbar.

Die PubMed-Recherche mit den Begriffen „ginger and rheumatoid arthritis“ ergab 73 Beiträge, für „ginger and spondyloarthritis“ wurden 5 und für „ginger and lupus“ 7 Ergebnisse gefunden, die keinen Bezug zu den jeweiligen Krankheitsbildern haben. 106 Zitate fanden sich für „ginger and osteoarthritis“.

Ingwerextrakte zeigten an Synovialzellkulturen vergleichbare antiinflammatorische Effekte wie Betamethason [43] und unterdrückten die Expression von TNF‑α und COX‑2 sowie die Synthese von Prostaglandin E2 [44] und Chemokinen [45]. Ein weiterer Inhaltsstoff des Ingwers, das Zingeron, hemmte in vitro den p38- und JNK-Signalweg, der für den Knorpelabbau bei Osteoarthrose bedeutsam ist [46]. 6‑Shogaol wirkte antientzündlich durch die Induktion von Apoptose und die Reduktion der Synthese von TNF‑α, IL‑6, und IL‑8 im Synovialgewebe [47]. In Tiermodellen senkte ein oral verabreichter Ingwerextrakt die Spiegel proinflammatorischer Zytokine (TNF‑α, IL‑6, IL-17) sowie die Expression von COX‑2 und NF-κB in Gelenkgewebe. Zudem wurden synoviale Entzündung, Hyperplasie und Knorpelabbau reduziert [48].

In einem Umbrella-Review zu den klinischen Wirkungen von Ingwer fanden sich 2022 Hinweise für schmerzlindernde Effekte bei Arthrose (Effektgröße: klein; Evidenzgrad: hoch). Die eingesetzten Dosierungen und der diätetische Verzehr waren sicher [49].

Eine iranische Arbeitsgruppe untersuchte 2019 die Wirkung von 750 mg Ingwerpulver 2‑mal täglich über 12 Wochen bei 66 RA-Patienten auf Laborvariablen und beschrieb u. a. eine Reduktion des CRP [50], lieferte jedoch keine klinischen Daten. Die gleiche Arbeitsgruppe untersuchte 2019 in einer randomisierten Doppelblindstudie an 70 RA-Patienten die Genexpression von Entzündungsmarkern. Die Verringerung des DAS28 war innerhalb der Ingwergruppe und zwischen den beiden Gruppen nach der Intervention statistisch signifikant [51].

Die Effekte von Ingwer bei Arthrose sind besser untersucht als bei RA. Eine Metaanalyse von 5 RCTs mit insgesamt 593 Patienten mit Kniearthrose zeigte für Ingwerextrakt signifikante Verbesserungen bei Schmerzen und Gelenkfunktion im Vergleich mit Placebo. Die eingesetzte Dosis war sehr heterogen (250–1000 mg Ingwerextrakt täglich, über 6–12 Wochen). Im direktem Vergleich zu NSAR war Ingwer in der Wirksamkeit unterlegen, aber besser verträglich [52]. Bei Gonarthrose-Patienten sanken bei Einnahme von 250–500 mg Ingwerpulver pro Tag über 6–12 Wochen die Zytokine (TNF‑α und IL-1β) sowie NO und CRP; diese Effekte hielten bis zu 12 Monate an [53, 54]. Klinische Parameter wurden nicht dokumentiert. Als Nebenwirkungen fanden sich lediglich milde gastrointestinale Beschwerden wie Reflux.

Im Jahr 2011 beschrieb eine placebokontrollierte Studie an 204 Gonarthrose-Patienten den Effekt von Ingwerextrakt über 6 Wochen. Die Ingwergruppe zeigte eine signifikante Reduktion von Schmerzen, Steifigkeit und funktionellen Einschränkungen bei identischen Nebenwirkungen [55]. Zuvor hatte eine doppelblinde placebokontrollierte Studie an 261 Gonarthrose-Patienten die Wirkung eines standardisierten Extrakts aus 2 Ingwersorten über 6 Wochen untersucht. Die Verumgruppe zeigte einen moderaten, aber signifikanten Effekt auf Schmerzen und WOMAC-Score bei gutem Sicherheitsprofil [56]. Zu ähnlichen Schlussfolgerungen kamen eine Arthrosestudie aus Korea mit erhitztem Ingwerextrakt [57], eine iranische Studie mit einem Sirup aus Ingwer und Honig („G-RUP“) [58] und eine italienische Studie mit einem Produkt aus Ingwer, Lecithin und Acmella oleracea (Jambu) [46, 59]. Ein Kombinationspräparat aus Ingwerextrakt und schwarzem Pfeffer unterschied sich in seiner Wirkung nicht von Naproxen und Ibuprofen in einer doppelblinden Studie [60]. Weitere Untersuchungen legten die Vergleichbarkeit von topischen Ingwerpräparaten mit anderen Externa wie Diclofenac-Gel, salicylathaltigen Salben oder mit einer Phonophorese nahe, wobei Ingwer als Monosubstanz und in Kombination getestet wurde. Dabei verursachten ingwerhaltige Salben in Einzelfällen eine lokale Dermatitis. Die aktuelle Gonarthrose-Leitlinie spricht ausdrücklich keine Empfehlung für oder gegen topische Phytotherapeutika inklusive Externa auf Ingwerbasis aus.

Es wurde vermutet, dass Ingwer das Blutungsrisiko bei gleichzeitiger Einnahme von Arzneimitteln wie Diclofenac oder Ibuprofen erhöhen kann. Da Ingwer die Thromboxanbildung und die Thrombozytenaggregation hemmen kann, könnte die gleichzeitige Einnahme von Antikoagulanzien das Blutungsrisiko erhöhen [61], obwohl die Datenlage dazu inkonsistent ist [62].

Die wissenschaftliche Evidenz reicht nicht aus, um die medizinische Anwendung von Ingwer bzw. Ingwerextrakten bei entzündlich-rheumatischen Erkrankungen zu empfehlen. Ein Ingwerpräparat mit definiertem Wirkstoffgehalt ist auf dem deutschen Arzneimittelmarkt nicht vorhanden. Da Ingwer aber auch andere günstige, vor allem metabolische Wirkungen hat, kann er in natürlicher Form für Patienten mit rheumatischen Erkrankungen als Nahrungsmittel empfohlen werden.

Für Arthrosen gibt es Evidenz von mäßiger bis mittlerer Qualität für eine leichte bis moderate schmerzlindernde bzw. antiinflammatorische Wirkung. Ingwerextrakte finden sich als NEM mit entsprechend geringem Qualitätsstandard auf dem Markt. Deren Einnahme kann nicht empfohlen werden. Wenn Patienten mit degenerativen Gelenkerkrankungen ergänzend zur etablierten Therapie Ingwer als Nahrungsmittel oder als qualitätsgesicherten Reinextrakt einnehmen möchten, muss aus rheumatologischer Sicht nicht abgeraten werden.

## Katzenkralle (Uncaria tomentosa)

Uncaria tomentosa (U.t., die Katzenkralle, englisch: „cat’s claw“, spanisch: „una de gato“) aus der Familie der Rötegewächse ist eine Kletterpflanze, die u. a. im Amazonasgebiet vorkommt. Dortige Ureinwohner wenden wässrige Extrakte aus der Rinde von Stamm und Wurzeln u. a. bei Arthritis, Asthma und Allergien an (Übersicht in [63]).

In Deutschland lassen sich über MMI Pharmindex Plus 7 Uncaria-Präparate aufrufen, davon 3 als NEM. In der Gelben Liste (www.gelbe-liste.de) sind 8 weitere Präparate (Kapseln, Rindenpulver, Tee und Liquid) als nichtapothekenpflichtige NEM deklariert.

Die Wurzel der Katzenkralle enthält Oxindol-Alkaloide. Diese besitzen proliferations- und entzündungshemmende, antivirale, antituberkulöse und antioxidative Effekte [64]. In Stamm und Wurzel wurden Terpenoide, Sterole und Flavonoide [63] sowie Hydroxycarbonsäuren [65] nachgewiesen. Innerhalb der Spezies existieren offenbar erhebliche Unterschiede im Wirkstoffgehalt. Letzterer wird zudem durch die Art der Ernte, der Lagerung und der Extraktion beeinflusst [66].

Eine PubMed-Recherche ergab zum Stichwort „uncaria tomentosa“ 129 Forschungsarbeiten, davon 16 unter den Stichworten „uncaria tomentosa and arthritis“.

Für U.t. sind wurden antientzündliche und immunmodulierende Wirkungen beschrieben. So hemmen Auszüge von U.t. die Apoptose von Lymphozyten im Tiermodell [67] und beinträchtigen die Aktivierung von mononukleären Zellen des peripheren Blutes (PBMC) durch Interferon gamma [68]. Ethanolische Rindenauszüge inhibierten in Zellkulturen TNF‑α durch die Hemmung von NF-κB [69, 70]. Entzündliche Pfotenödeme der Maus heilen durch alkoholische Auszüge von U.t. schneller ab als durch wässrige Extrakte [71]. Eine Mischung von Extrakten, in denen U.t. enthalten war, aber auch Boswellia und Peru-Ginseng, zeigte an Knorpelexplantaten und Chondrozyten eine entzündungshemmende und chondroprotektive Wirkung [72]. Extrakte von Uncaria guianensis unterdrückten die Wirkung von Interleukin‑1 auf Chondrozytenkulturen und Knorpelexplantate [73]. Die orale Behandlung der Adjuvansarthritis der Ratte mit wässrigen U.t.-Extrakten führte zu einer deutlichen Entzündungshemmung [74].

Klinische Studien zu U.t. bei rheumatologischen Indikationen sind spärlich. Bei Gonarthrosepatienten verbesserte gefriergetrockneter U.t.-Extrakt im Vergleich zu Placebo die globale Einschätzung von Arzt und Patient und den Bewegungsschmerz, hatte aber keine Auswirkungen auf Ruheschmerz und Knieschwellung [75]. Im Vergleich einer Mineralstoffmischung mit einer Kombination des Mineralstoffpräparates mit U.t. bei Arthrose bei 107 Patienten wurden keine Verbesserungen klinischer Parameter erreicht [76]. Für die RA existiert eine kleine Studie mit einem U.t.-Extrakt an 40 Patienten, in der Mehrzahl seronegativ. Im Vergleich zu Placebo verminderte die Gabe von U.t. in Kapselform signifikant die Zahl schmerzhafter, nicht jedoch die geschwollener Gelenke. Unbeeinflusst blieben die VAs für globale Krankheitsaktivität und globalen Schmerz und die Entzündungsparameter [77].

Die o. g. klinischen Studien berichteten eine gute Verträglichkeit von U.t. [75, 77]. Dennoch scheint die Anwendung nicht risikofrei zu sein. U.t.-Präparate sind in Zusammenhang mit einem Serotonin-Syndrom gebracht worden [78]. Die Verabreichung von U.t. hat zu Fällen interstitieller Nephritis geführt [79]. Ein Fall akuten Nierenversagens nach U.t.-Behandlung eines Patienten mit SLE wurde veröffentlicht [80]. Zudem wurde die Verschlechterung von motorischen Parkinson-Symptomen nach Einnahme von U.t. beschrieben [81]. Interaktionen mit Arzneimitteln sind nicht ausgeschlossen [82].

Die vorliegende Evidenz reicht nicht aus, um die Anwendung von U.t.-haltigen Zubereitungen für rheumatologische Indikationen zu empfehlen. Präparate mit eindeutig charakterisierter Zubereitung sowie definiertem und standardisiertem Wirkstoffgehalt sind in Deutschland nicht verfügbar. Von einer Selbstmedikation sollte zum jetzigen Zeitpunkt daher abgeraten werden. Auch wenn schmerzlindernde Effekte von Inhaltsstoffen von U.t. nicht ausgeschlossen werden können, gibt es keine Hinweise auf echte entzündungshemmende oder krankheitsmodifizierende Wirkungen. Daher sollte auf besser charakterisierte Alternativen zurückgegriffen werden.

## Knoblauch

Knoblauch (Allium sativum L) gehört zur Gruppe der Liliengewächse. Als Gemüse und Würzmitte wird er weltweit verwendet. Knoblauch wurde bereits in der altindischen Medizin als Mittel gegen „Rheuma und Unterleibsbeschwerden“ eingesetzt [83]. Genutzt werden Knoblauchöl zur äußeren Anwendung oder die frische und getrocknete Zwiebel.

Allicin (Allyl 2‑propenethionsulfinat oder Diallylthiosulfinat) als der wichtigste bioaktive Bestandteil kann sich in wässerigem Milieu spontan zu Allylsulfiden und Diallylsulfiden umwandeln. In Knoblauchölmazeraten entstehen aus Allicin schwefelorganische sekundäre Pflanzenstoffe, Vinyldithiine, Sulfide und Ajoene [83].

Der MMI Pharmindex plus listet über 30 verschiedene Knoblauchpräparate auf. Darunter finden sich Fertigpräparate wie Knoblauchtabletten oder -dragees, aber auch Mischpräparate mit Weißdorn oder Petersilie, Weißdorn und Mistel sowie Knoblauchöl und -salbe. Knoblauch wurde eine Vielzahl von relevanten Wirkungen zugesprochen – er soll u. a. lipid- und blutzuckersenkend, antioxidativ, analgetisch und antientzündlich wirksam sein [83, 84].

Die entzündungshemmenden und immunmodulatorischen Eigenschaften von Knoblauch und seinen Derivaten wurden in In-vitro- und Tiermodellen untersucht. Die schwefelorganischen Verbindungen des Knoblauchs stimulieren Makrophagen, Lymphozyten, natürliche Killerzellen, dendritische Zellen und Eosinophile durch die Modulation von Zytokinsekretion, Immunglobulinproduktion und Phagozytose. Unter den schwefelorganischen Verbindungen des Knoblauchs sticht Diallyltrisulfid (DATS) als potenziell entzündungshemmend hervor, indem es über den NF-κB-Signalweg die Transkription proinflammatorischer Zytokingene hemmt und die Bildung reaktiver Sauerstoffspezies reduziert [85].

Eine PubMed-Suche zum Stichwort „garlic“ ergab 8824 Treffer, zu „garlic and rheumatic diseases“ fanden sich 25 Literaturstellen. Für die Indikation „metabolisches Syndrom“ liegen Ergebnisse aus 16 RCT-Studien mit 999 Teilnehmern vor, die auf eine präventive bzw. therapeutische Wirkung von Knoblauch hinweisen [86]. Eine Metaanalyse von 16 RCTs über die Auswirkungen von Knoblauch auf entzündliche Biomarker überwiegend bei metabolischen Erkrankungen fand nur bei 3 von 13 auswertbaren Studien eine signifikante Verminderung des CRP. In der gepoolten Analyse war jedoch die Abnahme gegenüber Placebo signifikant, vor allem bei erhöhtem Ausgangs-CRP, bei Dosierungen von über 1200 mg Knoblauchpulver, einer Studiendauer über 12 Wochen und bei größerer Stichprobenzahl [87].

Zur mittelschweren bis schweren RA liegen zwei Arbeiten über die Ergebnisse einer doppelblinden RCT bei 70 Frauen vor, die 1000 mg Knoblauchpulver in Kapseln erhielten. Unter Knoblauch nahmen der DAS28, Schmerz-VAS, die Anzahl der schmerzhaften und geschwollenen Gelenke sowie das CRP im Vergleich zu Placebo signifikant ab [88, 89], ebenso der HAQ [88].

Eine Querschnittsstudie an einer großen Kohorte von 1086 weiblichen Zwillingen untersuchte den Einfluss von Ernährungsmustern auf die Entstehung einer Coxarthrose. Ein hoher Anteil an Obst und Gemüse wies einen umgekehrten Zusammenhang mit einer Coxarthrose auf (*p* = 0,022). Der Verzehr Lauchgewächsen war signifikant negativ assoziiert [90].

Eine bei 43 Patienten mit Gonarthrose durchgeführte 8‑wöchige RCT-Studie verglich die Wirkung eines multimodalen Rehabilitationsprogramms mit einer kombinierten Knoblauchtherapie (900 mg/Tag). Nach einer 8‑wöchigen Intervention kam es nur in der Knoblauchgruppe (*n* = 28) zu einem signifikanten Rückgang der synovialen Zytokine, einschließlich IL-1β, IL‑6 und TNF‑α. Dies ging einher mit einer besseren Kontrolle der Knieschmerzen und des Ergusses sowie mit einer besseren Muskelkraft und Lebensqualität. Die Studie liegt bisher nur als Poster vor [91]. In einer anderen Studie wurde die Wirkung einer Knoblauchsupplementierung (1000 mg/Tag) auf die Symptome bei 76 postmenopausalen übergewichtigen Frauen mit Gonarthrose untersucht. Nach 12-wöchiger Therapie verbesserten sich der WOMAC-Score nur in der Knoblauchgruppe signifikant, mit Ausnahme der Schmerzunterskala, die auch in der Placebogruppe abnahm. Signifikante Unterschiede zwischen den beiden Gruppen entstanden nicht [92]. Dieselbe Autorengruppe untersuchte die Auswirkungen einer Knoblauchsupplementierung auf das proinflammatorische Adipozytokin Resistin, auf TNF‑α und die Schmerzintensität. Nach der Intervention waren die Serumkonzentrationen von Resistin, nicht jedoch von TNF‑α in der Knoblauchgruppe signifikant gesunken, ebenso die Schmerz-VAS [93].

Knoblauch verändert den Geruch des Atems und der Haut. Die Einnahme kann Sodbrennen und andere gastrointestinale Beschwerden auslösen. Allergisches Asthma und Hautreaktionen sind nach Knoblaucheinnahme beschrieben [94]. Eine Allergie scheint verstärkt bei Menschen aufzutreten, die unter einer Pollenallergie leiden. Knoblauch könnte die Wirkung von Saquinavir, einem HIV-Medikament, abschwächen [95].

Auch wenn die vorliegenden Studien darauf hinweisen, dass Knoblauch oder Knoblauchpräparate einen die konventionelle Therapie unterstützenden Effekt bei RA und der Gonarthrose haben könnten, reicht die wissenschaftliche Evidenz nicht aus, um die Anwendung von Knoblauchextrakten für Symptome aufgrund eines definierten entzündlich-rheumatischen Krankheitsbildes zu empfehlen.

Da Knoblauch in der mediterranen Küche regelmäßig und in größerer Menge zum Einsatz kommt, kann Knoblauch in natürlicher Form für Patienten mit rheumatischen Erkrankungen als Nahrungsbestandteil empfohlen werden – dies insbesondere im Hinblick auf positive Effekte auf häufige Komorbiditäten bei rheumatischen Erkrankungen.

## Kohlblätter

Brassica oleracea var. capitata ist die botanische Bezeichnung für Kopfkohl, auch bekannt als Weißkohl oder Rotkohl. Kohl gehört zur Familie der Kreuzblütler (Cruciferae). Er wird entweder roh verzehrt oder verarbeitet, hauptsächlich durch Kochen oder Fermentieren. Die Form des Kohlblatts erleichtert das Auflegen auf die Haut in Form von Umschlägen (sog. Kohlwickel); Beschreibungen über Kohlwickel bei Schmerzen reichen bis zu Hildegard von Bingen zurück. Der Wickel bleibt dabei 1 bis zu 12 h auf dem betroffenen Gelenk. Die topische Anwendung soll durch ihre entzündungshemmenden, juckreizstillenden und antibakteriellen, aber auch kühlenden und abschwellenden Eigenschaften wirksam sein. Kohlwickel (aus Wirsing und Weißkohl) kommen in der Naturheilkunde bei Gelenkschmerzen, Arthrosen, Gichtanfällen, Sehnenscheidenentzündungen, Prellungen/Verstauchungen zum Einsatz.

Kohl enthält viel Vitamin C sowie die Vitamine A, B, E und K, dazu Kalzium, Eisen und Magnesium und Ballaststoffe. Die pharmakologischen Eigenschaften des Kohls werden durch Glucosinolate (Senföle), aber auch Flavonoide und Anthocyane erklärt. Kohl enthält zudem antioxidative phenolische Verbindungen. Die Zusammensetzung der Phytochemikalien hängt von der Art des Kohls, Anbauregion, klimatischen Bedingungen und Zubereitungsart ab. Die topische und systemische Verabreichung zeigte im Rattenmodell entzündungshemmende Wirkungen, die mit nichtsteroidalen entzündungshemmenden Medikamenten vergleichbar waren [96].

Eine PubMed-Suche nach wissenschaftlichen Beiträgen zum Stichwort „cabbage wraps“ ergab 27 Treffer, zu „cabbage wraps and rheumatic diseases“ fand sich 1 Literaturstelle. Zu RA gibt es keine Studien, zur Gonarthrose wurden 2 RCTs sowie ein narrativer Review publiziert [97]. In einer Studie wurden 81 Patienten mit einer Gonarthrose randomisiert einer vierwöchigen Behandlung mit Kohlwickeln, topischem Diclofenac-Schmerzgel oder mit „usual care“ (UC) aus Physiotherapie und Medikamenten zugeteilt. Nach 4 Wochen berichteten die Patienten der Kohlwickel-Gruppe über signifikant weniger Schmerzen als die UC-Gruppe. Im Vergleich zur Diclofenac-Gel-Gruppe fand sich kein signifikanter Unterschied. Kohlwickel wirkten signifikant besser bei einzelnen WOMAC-Skalen und Funktionstesten im Vergleich zur UC-Gruppe. Die Anwendungen wurden gut vertragen [98].

In einer weiteren nichtverblindeten Studie wurden 60 Patienten mit mittelschwerer bis schwerer Gonarthrose eingeschlossen. Die Teilnehmer wurden in 3 Interventionsgruppen eingeteilt: die Kühlgel-Pad-Gruppe (20 min Dauer einmal täglich), die Diclofenac-Gel-Gruppe (4-mal täglich) und die Kohlwickel-Gruppe (1 h Dauer einmal täglich). Die Anwendung von Kohlwickel und von kühlenden Gelkissen zeigten ähnliche Verbesserungen bei der Verringerung der OA-Symptome in Bezug auf den NRS-Gesamtwert (Schmerz) und den Oxford Knee Score (Funktion und Schmerz). Die therapeutische Wirksamkeit war besser als die von Diclofenac-Gel [99].

Die wissenschaftliche Evidenz reicht nicht aus, um die Anwendung von Kohlwickel für Patienten mit RA zu empfehlen. Bei ausdrücklichem Wunsch von Patienten mit Gonarthrose nach einer Alternative zur medikamentösen Therapie können Kohlwickel als Möglichkeit genannt werden. Dabei sollte auf die fehlende Standardisierbarkeit der Behandlung und die schwache Evidenz hingewiesen werden.

## Mariendistel

Die Mariendistel (Silybum marianum L. Gaernt.), auch wilde Artischocke genannt, ist eine Heilpflanze aus der Familie der Korbblütler (Asteraceae). In der Antike und in der traditionellen indischen und chinesischen Medizin wurde *Silybum marianum *zur Behandlung von Leberfunktionsstörungen eingesetzt. In Deutschland sind einige standardisierte Mariendistelpräparate verfügbar.

Der aus der Mariendistel gewonnene Extrakt Silymarin ist ein komplexes Gemisch aus Flavonolignanen, Flavonoiden sowie polyphenolischen Substanzen [100]. Die 4 Hauptisomere der Flavonolignane in Silymarin sind Silibinin, Isosilibinin, Silichristin und Silidianin. Unter diesen macht Silibinin etwa 50–60 % des gesamten Silymarin-Komplexes aus.

Silibinin zeigte in vitro in Zellmodellen die Fähigkeit zum Abfangen reaktiver Sauerstoffspezies [101, 102]. Zudem hemmt es Superoxid- und Stickstoffmonoxidradikale in Kupffer-Zellen [103]. Silymarin hemmt die Expression von TNF‑α, Interferon gamma, Interleukin(IL)-2 und IL‑4 durch die Blockierung des hepatischen NFκB [104]. Auch immunmodulatorische Effekte wie die Hemmung der NF-κB-vermittelten Aktivierung von CD4⁺-T-Zellen oder die Induktion eines ausgeprägten G1-Zellzyklusarrests in aktivierten T‑Lymphozyten wurden beschrieben. Silibinin und Silymarin zeigen antifibrotische Wirkungen in Tier- und In-vitro-Modellen der hepatischen Fibrogenese [105].

Eine Studie untersuchte die Wirkung von Silibinin (SIL) bei MTX-induzierter Hepatotoxizität in Ratten mit Adjuvans-induzierter Arthritis. Silibinin linderte die systemischen Auswirkungen der Arthritis, reduzierte Gelenkschäden und verhinderte histopathologische Veränderungen der Leber [106].

Eine PubMed-Recherche mit dem Suchbegriff „milk thistle and rheumatoid arthritis“ lieferte 4 Ergebnisse. Eine iranische nichtrandomisierte Studie mit 57 RA-Patienten, die ein Silymarin-Präparat (140 mg 3‑mal täglich über 3 Monate) als Ergänzung zur DMARD-Therapie erhielten, zeigte eine signifikante Besserung des DAS28-Score [107]. Es gab jedoch keine Vergleichsgruppe; ausgeschlossen wurden Patienten mit hoher Krankheitsaktivität, und die Basismedikation konnte bei anhaltender Krankheitsaktivität nicht verändert werden. Silymarin hatte keine Auswirkungen auf die Spiegel von TNF‑α und IL-1β bei RA-Patienten.

Ein aktueller systematischer Review von 2024 schlussfolgert, dass Silymarin und Silibinin vielversprechende Wirkungen auf RA- und OA-Symptome zeigten, aber weitere klinische Studien erforderlich seien, um eine klinische Verabreichung dieser Substanzen zu begründen [35, 108]. Den Effekt auf die OA haben die Autoren mit zwei irakische Studien begründet, in denen der Knee Injury and Osteoarthritis Outcome Score (KOOS) und die Serum Level von IL-1α und IL‑8, C3 und C4 herangezogen wurden. Silymarin konnte Leber- und Nierenfunktion bei Kindern mit ALL verbessern, die eine MTX-basierte Chemotherapie erhielten, aber die Autoren fordern weitere Studien, um eine protektive Wirkung zu belegen [109].

Hohe Dosen von Silymarin können zur Erhöhung des Bilirubins und der ALAT führen. Das Risiko klinisch relevanter Arzneimittelinteraktionen mit Silymarin wird als gering eingeschätzt. Dennoch sollten mögliche Wechselwirkungen bei gleichzeitiger Einnahme mit Substraten beachtet und überwacht werden, die über CYP450 verstoffwechselt werden. Obwohl Silymarin in vitro entzündungshemmende Eigenschaften aufweist, gibt es derzeit keine ausreichende Evidenz dafür, dass Mariendistelpräparate eine wirksame Behandlung für rheumatische Erkrankungen darstellen. Daher kann keine Empfehlung zur Anwendung ausgesprochen werden. Ob Mariendistelpräparate geeignet sind, hepatotoxische Effekte von Basistherapeutika wie MTX abzuschwächen oder zu verhindern, ist anhand der vorliegenden Daten nicht zu belegen.

## Pycnogenol

Pycnogenol ist ein Extrakt aus der Rinde der mediterranen See-Kiefer (Pinus pinaster), dem antioxidative und entzündungshemmende Eigenschaften zugeschrieben werden. Der MMI-Pharmindex listet 18 Präparate als nicht-apothekenpflichtige NEM auf, die zwischen 30 und 100 mg Pycnogenol enthalten.

In einer PubMed-Recherche wurde zur RA keine relevante Publikation gefunden, dafür einzelne Studien zur Sjögren-Erkrankung, systemischem Lupus erythematodes (SLE), Lupusvaskulitis und Morbus Behçet [110–114]. Eine Studie zur Sjögren-Erkrankung berichtet von einer signifikanten Reduktion der Blutsenkungsgeschwindigkeit und einer Verbesserung klinischer Symptome [113]. In einer offenen Registerstudie von Patienten mit SLE, Morbus Behçet, Sjögren-Erkrankung und Polyarteriitis nodosa wurde eine signifikante Reduktion von IL‑6 sowie eine verminderte Einnahme von Glukokortikoiden und nichtsteroidalen Antirheumatika in der Pycnogenol-Gruppe festgestellt [110]. In weiteren Studien an Patienten mit SLE und Vaskulitis wurde eine Verbesserung von Photosensibilität, oralen Ulzerationen und signifikant niedrigere Autoantikörpertiter gegen dsDNS sowie weniger Thrombozytopenien dokumentiert [111]. Allerdings ist unklar, ob die Patienten den jeweiligen Klassifikationskriterien entsprachen [111]. Zudem wurde in einer Studie eine signifikante Abnahme von oxidativem Stress und der Krankheitsaktivität bei SLE festgestellt [114]. Bei Studien zur Gonarthrose konnte bei kurzer Behandlungsdauer eine signifikante Reduktion der Schmerzen [115–117] und der Einnahme von Schmerzmedikamenten [115, 116] festgestellt werden. Bei Arthrose der Hand verbesserten sich Schmerzen und Griffstärke [118]. Die Studien weisen signifikante methodische Defizite auf. Die Untersuchungen sind offen, unkontrolliert und basieren auf kleinen Fallzahlen. Vergleichsstudien mit etablierten Medikamenten sind nicht vorhanden. Auf Nebenwirkungen wird nicht eingegangen.

Für den Einsatz von Pycnogenol zur Behandlung von entzündlich-rheumatischen Erkrankungen existieren derzeit keine ausreichenden Daten. Bei ausdrücklichem Wunsch von Patienten mit Arthrosen nach einer phytotherapeutischen Ergänzung zu einem etablierten Therapiekonzept kann Pycnogenol als Möglichkeit der Selbstmedikation genannt werden. Dabei sollte auf die schwache Evidenz hingewiesen werden.

## Schachtelhalm

Schachtelhalme sind die einzige Gattung in der Pflanzenklasse der Equisetopsida innerhalb der Farne. Der Acker-Schachtelhalm (Equisetum arvense) hat eine Historie als Heilpflanze. Mindestens 229 chemische Substanzen konnten aus dieser Pflanze isoliert werden, die meisten davon Flavonoide. Diese Substanzen haben potenziell antimikrobielle, antiinflammatorische, antidiabetische, hepatoprotektive Fähigkeiten und sind Antioxidanzien [119, 120]. Traditionell wurde der Schachtelhalm zur lokalen Wundversorgung und bei „Rheuma“ eingesetzt.

Im MMI Pharmindex wurden zu Schachtelhalm 11 Wirkstoffe und Gruppen gefunden, darunter Mischextrakte, z. B. aus Eibischwurzel, Eichenrinde, Kamillenblüten, Löwenzahnkraut, Schachtelhalmkraut, Schafgarbenkraut und Walnussblättern. Dazu kommen Extrakte, Pulver, getrocknete Pflanzenteile und Presssaft, entweder als externe Pflegemittel oder NEM. Vier Präparate sind apothekenpflichtig. Standardisierte Präparate sind nicht verfügbar.

Eine PubMed-Recherche erbrachte zu „Horsetail herb“ 6 Referenzen, eine mit „Rheuma*“, zu „Equisetum“ 555 Zitate, davon 2 mit „Rheuma*“ und 7 mit „Arthritis“.

Als eine potenziell wirksame Substanz des Schachtelhalms wird KYNA („kynurenic acid“) benannt [121]. Die KYNA-Spiegel sind bei Patienten mit unbehandelter RA erniedrigt [122]. Ein Einsatz in einer Studie ist hierzu nicht publiziert.

Eine Studie an 6 Ratten mit Adjuvans-induzierter Arthritis konnte eine dosisabhängige Besserung klinischer Befunde, Histologie und Zytokinspiegel durch Equisetum-diffusum-Methanol-Extrakt (EDME) zeigen [123]. Im Antigen induzierten Maus-Arthritismodell konnte ein Extrakt von Equisetum giganteum zahlreiche Surrogatparameter verbessern [124]. In vitro hemmte ein Equisetum-arvense-Extrakt die Lymphozytenproliferation und -aktivierung sowie die Zytokinproduktion [125].

Bei Pferden kann der Verzehr von Schachtelhalm zur sog. Taumelkrankheit führen. Auch bei Menschen wurden toxische Wirkungen berichtet.

Es liegen keine Studien zum Einsatz beim Menschen vor. Eine Übertragung der Daten von Arthritis-Tiermodellen auf die RA ist nicht gerechtfertigt. Aufgrund der Datenlage kann der Einsatz von Schachtelhalm bei entzündlich-rheumatischen oder degenerativen Erkrankungen nicht empfohlen werden.

## Teufelskralle

Die Afrikanische Teufelskralle (*Harpagophytum procumbens*), auch Trampelklette genannt, stammt aus der Familie der Sesamgewächse (Pedaliaceae). Sie wird in der traditionellen afrikanischen Medizin bei muskuloskeletalen Symptomen (z. B. durch Osteoarthrose) und bei Rückenschmerzen eingesetzt. Aus ihren Wurzelknollen werden u. a. Pulver, Aufgüsse, Tinkturen und Extrakte hergestellt [126]. Eine entzündungshemmende und schmerzlindernde Wirkung wird dem Iridoidglykosid Harpagosid zugeschrieben [127]. In In-vitro- und Ex-vivo-Studien verhinderte es die Freisetzung von TNF-α- und IL‑6 sowie die Produktion der Enzyme COX‑2 und iNOS [128, 129]. Antioxidative Eigenschaften von Flavonoiden und Phenolen tragen zur Reduktion von oxidativem Stress bei [130]. Außerdem hemmt Harpagosid die Osteoklastenbildung in vitro und könnte so den entzündungsbedingten Knochenabbau unterdrücken [131]. Weitere Bestandteile der Teufelskralle sind Phenylethanolglykoside, Phytosterine, Triterpene und Zimtsäure. Die Bioverfügbarkeit und Stabilität der Wirkstoffe im menschlichen Körper sind nicht bekannt.

In Deutschland sind etwa 115 Präparate mit Teufelskralle v. a. als NEM und als Nichtarzneimittel verfügbar, einige auch als pflanzliche rezeptfreie, apothekenpflichtige Arzneimittel. Darunter finden sich Tropfen, Homöopathika und Filmtabletten mit Teufelskrallenwurzel-Trockenextrakt, entweder ohne eindeutige Anwendungsbezeichnung oder „bei Erkrankungen des Bewegungs- und Stützapparates“ bzw. „zur unterstützenden Behandlung bei Verschleißerscheinungen des Bewegungsapparates.“.

Eine PubMed-Suche nach wissenschaftlichen Beiträgen zum Stichwort „devils claw“ lieferte 221 Literaturstellen, zum Stichwort „Harpagophytum procumbens“ 106 Literaturstellen. Davon sind 65 klinische Studien. Diese betreffen überwiegend nicht-entzündliche rheumatologische Indikationen wie neurologische Erkrankungen sowie degenerative Gelenk- und Wirbelsäulenveränderungen (als unspezifische Rückenschmerzen, Gonarthrose etc.). Hingegen sind zu entzündlich-rheumatischen Erkrankungen keine klinischen Studien vorhanden.

Daten zur Wirksamkeit der Teufelskralle liegen aus Studien zu Osteoarthrose vor: Ein narrativer Review von 12 klinischen Studien (8 davon placebokontrolliert, 4 mit anderen Therapien im Vergleichsarm) konstatiert eine moderate Evidenz für die Wirksamkeit eines Pulvers mit 60 mg Harpagosid bei der Behandlung von degenerativen Veränderungen der Wirbelsäule sowie des Hüft- und des Kniegelenks. Eine moderate Evidenz fand sich ebenso bei der Verwendung eines wässrigen Extrakts mit einer Tagesdosis von 100 mg Harpagosid bei akuten Exazerbationen chronischer unspezifischer Rückenschmerzen. Bei der gleichen Indikation waren ein wässriger Extrakt aus mit 60 mg Harpagosid und 12,5 mg Rofecoxib pro Tag vergleichbar wirksam [132].

Eine Übersichtsarbeit zum Einsatz von Teufelskralle bei Arthrose berücksichtigte 14 Studien von 1966 bis 2006, darunter vier doppelblinde, placebokontrollierte, randomisierte Studien. Diese zeigten vielfach eine unzureichende Qualität. Die hochwertigeren Studien deuteten jedoch auf eine schmerzlindernde Wirkung hin [133]. In einer Studie wurde die Wirksamkeit von der Teufelskralle (2 × 480 mg) über einen Monat im Vergleich zu Meloxicam (15 mg) über 10 Tage bei 60 Patienten mit Gonarthrose untersucht. Der WOMAC-Index verbesserte sich in beiden Gruppen [134]. Eine multizentrische, doppelblinde Studie an 122 Patienten mit Hüft- und/oder Kniearthrose verglich Harpagophytum 2610 mg pro Tag mit Diacerein 100 mg pro Tag. Nach 4 Monaten zeigten sich in beiden Gruppen deutliche und zwischen den Gruppen vergleichbare Verbesserungen von Schmerzen, funktioneller Behinderung und des Schmerzmittelgebrauchs [135]. In einer dreiarmigen Doppelblindstudie an 197 Patienten mit chronischen unspezifischen Rückenschmerzen wurden über 4 Wochen 50 bzw. 100 mg Harpagosid mit Placebo verglichen. Die Anzahl der schmerzfreien Patienten betrug bei Placebo 5 % sowie 10 % und 16 % in der niedrigen bzw. hohen Verumgruppe [136]. Eine Cochrane-Analyse zur Wirksamkeit von Phytotherapeutika bei unspezifischen Rückenschmerzen zitierte neben der vorherigen Studie drei weitere von geringer oder sehr geringer Qualität [137]. Eine doppelblinde Studie applizierte bei Patienten mit Muskelschmerzen im Rücken‑, Schulter- und Nackenbereich entweder 2 × 480 mg/Tag Harpagosid-Extrakt oder Placebo. Nach 4 Wochen zeigte sich eine Wirksamkeit des Verums im Gesamtscore, in den Patienten- und Arztbewertungen, dem Druckalgometertest und dem Muskelsteifigkeitstest [138].

Die Anwendung von Teufelskralle wird als gut verträglich beschrieben, es gibt aber Nebenwirkungen, die berücksichtigt werden sollten. In einer Literaturrecherche zur Sicherheit von Hp-Präparaten wurden in 20 von 28 klinischen Studien von Nebenwirkungen berichtetet. In keiner der Doppelblindstudien waren Nebenwirkungen unter Teufelskralle häufiger als unter Placebo. Leichte Nebenwirkungen bei 3 % der Patienten betrafen Übelkeit, Durchfall oder Bauchschmerzen. Es gab einige Berichte über akute, jedoch keine Berichte über chronische Toxizität [139]. Teufelskralle scheint nach vorläufigen Daten ein geringeres Nebenwirkungsrisiko als NSAR zu haben [133]. Im Beipackzettel für Teufelskralle werden Magen-Darm-Beschwerden sowie Schwindel und Kopfschmerzen aufgeführt. Überempfindlichkeitsreaktionen bis zum anaphylaktischen Schock sind beschrieben worden. Bei insulinpflichtigem Diabetes mellitus wurde ein vorübergehender Blutzuckeranstieg beobachtet. Von einer Anwendung in Schwangerschaft und Stillzeit wird mangels ausreichender Daten abgeraten. Zusammenfassend ist die wissenschaftliche Evidenz eingeschränkt. Die verfügbaren Studien haben in der Regel kleine Stichprobengrößen, es fehlen Daten zur Sicherheit bei langfristiger Anwendung, insbesondere bei höheren Dosierungen. Die Konzentration des Wirkstoffs Harpagosid variiert zwischen den Produkten, was die Vergleichbarkeit der Studien erschwert.

Die Effekte von Bestandteilen aus Harpagophytum auf Entzündungsmediatoren in vitro und in vivo schließen ein therapeutisches Potenzial nicht aus. Systematische klinische Studien der Harpagophytum bei entzündlich-rheumatischen Krankheitsbildern sind nicht vorhanden, die Anwendung zur Therapie dieser Erkrankungsgruppe kann daher derzeit nicht empfohlen werden. Bei ausdrücklichem Therapiewunsch von Patienten mit unspezifischen Rückenschmerzen muss von der Anwendung von Teufelskralle-Präparaten nicht abgeraten werden.

## Weihrauch (Boswellia)

Weihrauch ist das Harz des Boswellia-Strauches, vor allem der Boswellia serrata. Für medizinische Zwecke verwendeter Weihrauch wird auch Olibanum genannt und oral eingenommen. Er ist ein Bestandteil der ayurvedischen Medizin.

Als über die Prostaglandin- und Leukotrien-Synthesehemmung entzündungshemmende Bestandteile des Weihrauchharzes gelten 11-keto-β-boswellic acid (KBA) und acetyl-11-keto-β-boswellic acid (AKBA) [140, 141]. Ihnen wird eine Hemmung von Metalloproteinasen [142], TNF-α- und NF-κB-Hemmung [143, 144], Stabilisierung der (Lip‑)Oxygenasen [145] sowie der extrazellulären Matrix zugeschrieben [146].

Kombinationen von Weihrauchharzen mit MTX in hohen Dosen wurden in vitro [147] und im Tiermodell [148] getestet. Zur Besserung der Bioverfügbarkeit wurden tierexperimentelle Untersuchungen mit Weihrauch-NSAR-Hybriden [149] und Nanopartikeln [150] vorgestellt.

Im MMI sind Indischer Weihrauchbaumharz-Extrakt, Weihrauch, Weihrauch-Extrakt und Weihrauch-Öl gelistet. Diese werden in 300 Präparaten überwiegend als NEM vertrieben. Apothekenpflichtig ist 1 Produkt. In der „Roten Liste“ sind 8 Produkte als Homöopathika gelistet.

Eine PubMed-Suche zum Stichwort „Boswellic acid“ lieferte 640 Literaturstellen, davon 48 mit gleichzeitiger Nennung „Arthritis“. Trotz zahlreicher Publikationen zur Besserung von Surrogatparametern in vitro und im Tiermodell einer Adjuvans-induzierten Arthritis sind nur wenige Studien zur Wirksamkeit des Weihrauchharzes am Menschen veröffentlicht worden.

In Deutschland wurden zwei doppelblinde Studien mit dem Präparat H15 (Boswellinsäure) bis 3600 mg/Tag bei langjähriger RA durchgeführt und eine signifikant dem Placebo überlegene Wirkung als Abstract veröffentlicht [151]. Eine Neuberechnung der Rohdaten konnte einen Vorteil von Weihrauch nicht belegen [152].

Zur Gonarthrose existieren mehrere Untersuchungen. Jeweils 15 Patienten mit Gonarthrose wurden mit 333 mg Weihrauch oder Placebo für 8 Wochen im Cross-over-Design behandelt. Wirksamkeitsparameter waren VAS für Schmerz, Schwellung und Funktionseinschränkung. Unter Verum werden Besserungen aller 3 Parameter um 80–90 % benannt, unter Placebo um maximal 10 %. Diese in ihrer Ausprägung unglaubhafte Besserung konnte bei Therapiewechsel in der 2. Gruppe erneut berichtet werden [153]. Bei einer Nachuntersuchung nach 6 Monaten zeigte eine Gonarthrose-Behandlung über 2 Monate mit 7,2 mg Boswellinsäure/Tag einen tendenziellen Vorteil gegenüber Glucosaminen, eingeschlossen waren je Arm 60 Patienten [154]. 80 Patienten mit Gonarthrose, die über 180 Tage randomisiert 100 mg Weihrauch oder Placebo erhalten hatten, zeigten Besserungen von funktionellen Scores, von Laborwerten wie CRP sowie von Knorpeldicke und Gelenkspalt in der MRT mit sehr hohem Signifikanzniveau [155]. In einer Metaanalyse von 7 Studien mit 545 Patienten wird eine signifikanter Besserung von Schmerz und Funktion berichtet und Boswelliaharze wurden als effektiv und sicher bewertet [140]. Die Studienpopulationen sind jedoch klein und ein starker Untersucherbias ist anzunehmen. Daten zur Wirkung bei anderen Arthrosen liegen nicht vor. Die Besserungen von 80 % aller Parameter innerhalb von 3 Wochen und die hochsignifikante Zunahme der Knorpeldicke in der MRT, die sich den genannten Studien finden, sind so wenig plausibel, dass sie für eine Beurteilung der Wirksamkeit nicht genutzt werden können [153, 155]. Eine optimale Dosis ist nicht erkennbar.

Die Sicherheit der Anwendung von Weihrauch in den getesteten Dosierungen bis 3600 mg/Tag ist in den publizierten Studien hoch und vergleichbar mit der Placebotherapie.

Die wissenschaftliche Evidenz reicht nicht aus, um die Verschreibung und Anwendung von Weihrauchpräparaten für Patienten mit Symptomen aufgrund einer entzündlich-rheumatischen Krankheit zu empfehlen. Die Effekte von Bestandteilen aus Weihrauchpräparaten auf Entzündungsmediatoren in vitro und in Tier-Arthritismodellen erlauben keine Rückschlüsse darauf, dass entzündungshemmende Bestandteile des Harzes beim Menschen wirksam werden. Systematische kontrollierte klinische Studien an Fallkollektiven mit klar umrissenen rheumatologischen Krankheitsbildern konnten keinen Beleg einer Überlegenheit gegenüber Placebo zeigen.

Bei Behandlungswunsch der Gonarthrose auf Seiten der Patienten muss von einer Einnahme von Weihrauch nicht abgeraten werden.

## Wilfords Dreiflügelfrucht (Tripterygium wilfordii)

Tripterygium wilfordii (TW), eine Heilpflanze, die in der traditionellen chinesischen Medizin Anwendung findet, hat Aufmerksamkeit als potenzielle Therapieoption bei der rheumatoiden Arthritis (RA) erfahren.

In der vorliegenden Arbeit wurden ausschließlich Studien berücksichtigt, in denen Tripterygium wilfordii als Extrakt der ganzen Dreiflügelfrucht-Pflanze und nicht als Gemisch aus verschiedenen pflanzlichen Substanzen appliziert wurde. Insgesamt 13 Studien wurden in die Analyse integriert.

Der MMI Pharmindex listet unter dem Stichwort „Tripterygium“ ein einziges Präparat als Vollextrakt auf, welches als nichtapothekenpflichtiges NEM deklariert wird.

In Übersichtsarbeiten und Metaanalysen wurde die Wirksamkeit von TW in der Behandlung der RA im Vergleich zu konventionellen synthetischen DMARDs wie Methotrexat oder Sulfasalazin [156–158] dokumentiert. Tripterygium wilfordii verbesserte als Monotherapie und in Kombination klinische Parameter wie die Anzahl geschwollener oder schmerzhafter Gelenke und CRP signifikant [159, 160]. Das ACR-20-Ansprechen war besser als unter Sulfasalazin [6]. Bei Patienten mit einer systemischen Sklerose wurde die Verbesserung der forcierten Vitalkapazität und Lungenbeteiligung unter der Therapie mit Tripterygium wilfordii beschrieben [161]. Weitere Studien zu den anderen entzündlich-rheumatischen Erkrankungen fanden sich nicht.

Bei 46,4–48 % der mit TW behandelten RA-Patienten wurde mindestens eine Nebenwirkung beobachtet: Dabei wurden u. a. Leberfunktionsstörung (9,6–11,5 %), Übelkeit (13–22 %), Erbrechen (15 %), Durchfall (25 %), Infektionen der oberen Atemwege (1,9–18 %), Menstruationsstörungen (5,0–12,5 %) und Hautausschlag (12 %) beschrieben [159, 162–164].

Die Mehrzahl der Studien basierte auf schwachen Endpunkten wie der ACR20-Ansprechrate [156, 158]. Robustere Endpunkte wurden nur in Einzelfällen berichtet [160]. Die Bewertung der radiologischen Progression erfolgte in lediglich einer Langzeitstudie. Vergleichsstudien mit biologischen oder „targeted synthetic DMARDs“ fehlen. Ein zugelassenes Präparat mit definiertem Wirkstoffgehalt ist in Deutschland nicht vorhanden. Bei der Verwendung von Tripterygium wilfordii können unerwünschte Arzneimittelwirkungen, insbesondere Leberfunktionsstörungen und abdominelle Beschwerden, auftreten. Von einer Verwendung von TW als Nahrungsergänzungsmittel mit dem Ziel einer Linderung muskuloskeletaler Symptome wird abgeraten.

## Diskussion

Mit den hier vorgestellten pflanzlichen Präparaten ist die Liste der von der Kommission für Komplementäre Heilverfahren und Ernährung begutachteten Heilpflanzen und ihrer Zubereitungen auf nunmehr 21 angewachsen. Die darauf basierenden Empfehlungen bilden den aktuellen Stand ab und können sich selbstverständlich ändern, wenn neue Studien zu diesen und anderen pharmakologisch interessanten Pflanzen veröffentlicht werden. Die Aufstellung erhebt keinen Anspruch auf Vollständigkeit. Sie berücksichtigt auch nur Heilpflanzen, die in Deutschland traditionell angewendet werden, oder Substanzen wie Weihrauch und Teufelskralle, die in einer globalisierten Welt den Weg nach Europa gefunden haben. Weltweit bestehen große Unterschiede in der Verbreitung und Anwendung von Phytopharmaka oder pflanzlichen Präparaten. So finden in der chinesischen Medizin Extrakte der Pfingstrose („total glycosides of paeony“) für die rheumatoide Arthritis breite Anwendung und stoßen auf wissenschaftliches Interesse. Während diese Polyphenole in Deutschland weitgehend unbekannt sind, analysierte unlängst eine Metaanalyse 9 klinische Studien zu dieser Indikation – allesamt von chinesischen Autoren [165]. Sowohl die traditionelle chinesische Medizin als auch die ayurvedische Medizin berufen sich in starkem Maße auf die Verwendung von Heilpflanzen. Der bereits in unserer ersten Publikation [1] erwähnte Bericht einer gemeinsamen Expertenkommission des Bundesamts für Verbraucherschutz und Lebensmittelsicherheit und des BfArM zur Einstufung von Stoffen führte im Januar 2020 aus, dass allein in Indien ca. 7000 Pflanzen zur Therapie verschiedenster Erkrankungen genutzt werden und 700 in ayurvedischen Medizinsystemen gelistet sind^4^.

Diese Aufstellung ist nach wie vor gültig und wird aktualisiert. So ist für 2026 eine Stellungnahme zur Anwendung der Schlafbeere als Mittel der ayurvedischen Medizin zur Stressreduktion, Schlafförderung und Entzündungshemmung angekündigt sowie eine Stellungnahme zu den in der traditionellen chinesischen Medizin eingesetzten Substanzen.

Vor dem Hintergrund einer immer effektiveren Therapie entzündlich-rheumatischer Erkrankungen ist auch mit diesem zweiten Beitrag der Kommission klargeworden, dass der Stellenwert der Phytotherapie für entzündliche Gelenk- und Wirbelsäulenerkrankungen marginal bleibt. Nach dem besten Wissen der AutorInnen befinden sich auch keine phytotherapeutischen Präparate in der Entwicklungsphase, die an diesem geringen Stellenwert etwas ändern könnten.

Im Hinblick auf degenerative Erkrankungen und auf die Therapie schlecht eingrenzbarer Gelenk- und Wirbelsäulenschmerzen muss die Situation differenzierter dargestellt werden. Einerseits gilt hier das Prinzip des „primum nil nocere“, welches durch die Gabe von potenten NSAR oder gar Opiaten bei oft langanhaltenden muskuloskeletalen Schmerzen durchaus verletzt werden kann. In einem Zeitalter des „shared decision-making“ lässt sich der Wunsch von Patienten nach einer pflanzenbasierten Symptombehandlung nicht einfach mit dem Hinweis auf eine unzureichende Datenlage ablehnen. Dem Grundsatz „Absence of evidence is not evidence of absence“ hat die Kommission auch bei der zweiten Analyse Rechnung getragen, indem sie bei degenerativen Gelenkerkrankungen zwischen „keine Empfehlung“ und „bei Patientenwunsch wird nicht abgeraten“ differenziert hat. Mit dem Hinweis darauf, die Heilpflanzen Ingwer, Curcuma und Knoblauch nicht als (oft teure) NEM sondern als Nahrungsmittel anzuwenden, schlägt die Kommission zudem eine Brücke zwischen Phytopharmaka und einer pflanzenbasierten, antientzündlich ausgerichteten Ernährung. Da es nicht realistisch ist, auf Studien mit Phytopharmaka zu pochen, die höchsten Ansprüchen an wissenschaftliche Evidenz gerecht werden, scheint dieses Vorgehen dem Alltag in der rheumatologischen Praxis am ehesten gerecht werden zu können.

## Data Availability

Alle dieser Arbeit zugrunde liegenden Daten sind in diesem Artikel enthalten.

## Footnotes

1 Monographie BGA/BfArM (Kommission E) Erscheinungsdatum Bundesanzeiger: 27.07.1990., Heftnummer: 138., ATC-Code: D11AG.

2 https://register.awmf.org/assets/guidelines/033-001l_S2k_Koxarthrose_2019-07_1-abgelaufen.pdf.

3 https://www.bvl.bund.de/SharedDocs/Downloads/01_Lebensmittel/expertenkommission/Stellungnahme_Curcumin.pdf?__blob=publicationFile&v=2.

4 https://www.bfarm.de/SharedDocs/Downloads/DE/Arzneimittel/Zulassung/ZulRelThemen/abgrenzung/Expertenkommission/stellungnahmen/2020-01.pdf?__blob=publicationFile.
